# Thrombocytopenia and Epistaxis Complicating *Plasmodium falciparum* Malaria: Case Report Study

**DOI:** 10.1155/carm/6670368

**Published:** 2025-03-20

**Authors:** Mugahid A. Mobark

**Affiliations:** Department of Pharmacy Practice, College of Pharmacy, Qassim University, Buraydah 51452, Saudi Arabia

**Keywords:** epistaxis, malaria, *Plasmodium falciparum*, thrombocytopenia

## Abstract

Malaria is one of the vector-borne diseases with major public health problems to date, especially in endemic areas. Malaria is usually associated with various degrees of reduced red blood counts, and thrombocytopenia is a common association of malaria, but it is rarely associated with hemorrhagic manifestations. This case report presents two Sudanese patients, who presented to a private clinic in North Bahri. The patients were infected with *Plasmodium falciparum*; they developed thrombocytopenia and were presented clinically with epistaxis. A 36-year-old male and a 41-year-old male presented with the clinical features of malaria and epistaxis and were diagnosed with *P. falciparum* malaria. Both patients had significant thrombocytopenia in complete blood count (CBC). They showed clinical and laboratory improvement of thrombocytopenia after successful treatment of malaria. Although bleeding is a rare complication of malaria associated with thrombocytopenia, it should be considered even if it presents as a minor bleeding such as epistaxis or bleeding from gum.

## 1. Introduction

Malaria is a life-threatening disease caused by a *Plasmodium* parasite that is transmitted to humans through bites of the infected females of Anopheles mosquitoes [1]. *Plasmodium falciparum*, *Plasmodium vivax*, *Plasmodium ovale*, *Plasmodium knowlesi*, and *Plasmodium malariae* are the five species that cause malaria in humans. *P. falciparum* is the most dangerous of these, causing the most severe form of the disease and responsible for many deaths annually, primarily in Sub-Saharan Africa [2]. The clinical symptoms of malaria include the classic paroxysm of fever with chills and rigors followed by sweating and perspiration [3]. Diagnosis of malaria involves clinical evaluation and laboratory tests. Hyperparasitemia denotes a significantly elevated level of parasite in the blood and is commonly linked to severe complications of malaria, and hyperparasitemia is often seen with *P. falciparum* infections [4]. Several factors can contribute to hyperparasitemia, including delayed diagnosis and treatment, drug resistance, and impaired immune function [5]. Hyperparasitemia can lead to a cascade of severe complications, including severe anemia, organ dysfunction, coma, and death. The high parasite density leads to the extensive destruction of red blood cells, resulting in severe anemia [6]. Moreover, the accumulation of parasites and infected red blood cells can obstruct blood flow to vital organs, leading to dysfunction, particularly in the brain, kidneys, and lungs [7]. In severe cases, hyperparasitemia can lead to cerebral malaria, characterized by coma and seizures, which can be fatal [8]. Thrombocytopenia, a condition characterized by an abnormally low platelet count, is a common complication of malaria infection especially with infection [9]. The pathogenesis of thrombocytopenia in malaria is complex and more than one factor can play a role in its development. However, thrombocytopenia could be strongly related to splenomegaly, coagulation impairments, and damage to the platelets by macrophages; in addition to that, oxidative stress, distortion of the bone marrow, and aggregation of platelets also play a significant role [10].

Thrombocytopenia is divided into three classes: mild thrombocytopenia (platelet counts 50,000 to less than 150,000 cells/μL), moderate thrombocytopenia (platelet counts 20,000 to less than 50,000 cells/μL), and severe thrombocytopenia (platelet counts less than 20,000 cells/μL) [11].

Bleeding manifestations in malaria can range from mild to severe and are often associated with thrombocytopenia [12]. In this case report study, two cases of malaria are presented with hyperparasitemia presented to a private clinic in North Bahri in Khartoum with epistaxis.

## 2. Case Presentation

  Case 1. A 36-year-old male presented with intermittent fever, fatigability, nausea, and loss of appetite for two days. Two hours before arrival at the clinics, he started to develop epistaxis. On physical examination, the patient was fully conscious, looked ill, and his temperature was 39.2°C. He was slightly pale but not jaundiced. Blood pressure and other vital signs were normal. Investigations showed a positive ICT (immunochromatographic assay) for *P. falciparum*, and blood film for malaria showed *P. falciparum* with moderate parasitemia (+++). Blood urea, serum creatinine, sodium, and potassium were normal. The complete blood count (CBC) showed low RBCs, low hemoglobin, low hematocrit, low mean cell volume, low mean cell hemoglobin, and low mean cell hemoglobin concentration with significant low platelets' count (moderate thrombocytopenia) (Table 1).
  Case 2. A 41-year-old male presented with intermittent fever, muscle aches, nausea, abdominal pain, vomiting, and two attacks of epistaxis in one day. On physical examination, the patient was ill and febrile. The body temperature was 38.8°C. Blood pressure and other vital signs were normal. Investigations showed a positive ICT for *P. falciparum*, and blood film for malaria showed *P. falciparum* with moderate parasitemia (+++). Blood urea, serum creatinine, sodium, and potassium were normal. The CBC showed a slight decrease in hemoglobin, hematocrit, and mean cell hemoglobin with significantly low platelets' count (mild thrombocytopenia) (Table 1).

Local hemostatic management of epistaxis was performed for both patients, and every patient received 80 mg artemether and 480 mg lumefantrine (AL 80/480) twice a day for 3 days. The patients were followed, and after 3 days, both patients clinically improved and there was no epistaxis. Then, both patients were followed after 1 month and the CBC showed an improvement in RBC indices and platelets' count (Table 2).

## 3. Discussion

The occurrence of thrombocytopenia with *P. falciparum* malaria is reported in more than one study, and it is usually mild to moderate and very rarely symptomatic [13–15]. In this case report, thrombocytopenia with malaria is presented clinically with epistaxis. While bleeding is not a universal symptom of malaria, it can occur as a complication, particularly in severe cases. Several factors contribute to bleeding in malaria, including platelet destruction and disseminated intravascular coagulation (DIC), and in severe cases, malaria can trigger DIC, a condition characterized by widespread activation of the blood clotting system, which can paradoxically lead to both clotting and bleeding [12]. Bleeding in malaria can manifest as a minor bleeding in the form of easy bruising, petechiae, and bleeding from the gums or nose or it can manifest as severe bleeding in the form of gastrointestinal bleeding, bleeding into the brain, or bleeding from other sites. The risk of bleeding is higher in severe cases, particularly in those with *P. falciparum* infection and other complications such as hyperparasitemia [13, 16]. Although bleeding does not occurr frequently with malaria, its development specifically in endemic areas should raise the possibility of thrombocytopenia occurring with malaria and can even guide the suspicion of malaria infection.

## 4. Conclusion

Although bleeding does not occur frequently with malaria, this case report of thrombocytopenia and epistaxis complicating *P. falciparum* infection highlights the importance of considering the clinical presentation of bleeding, even minor ones such as epistaxis, especially in malaria-endemic areas.

## Figures and Tables

**Table 1:** The complete blood count (CBC) of Cases 1 and 2 preantimalarial treatment.

CBC	Case 1	Case 2	Reference range
White blood cells	5.3	6.5	4.0–11.0 × 10^9^/L
Red blood cells	4.2	4.5	4.5–6.5 × 10^12^/L
Hemoglobin concentration	9.7	12.1	12.5–18.0 g/dL
Hematocrit	32.0	34.1	40%–52%
Mean cell volume	73.2	78	78–100 fL
Mean cell hemoglobin	23.1	26.8	27–32 pg
Mean cell hemoglobin concentration	30.3	34.4	31–37 g/dL
Platelets	26	72	150–400 × 10^9^/L
Neutrophils	3.2	3.7	2–7.5 × 10^9^/L
Lymphocytes	1.8	2.1	1–4.5 × 10^9^/L
Monocytes	0.15	0.4	0.2–0.8 × 10^9^/L
Eosinophils	0.09	0.21	0.04–0.40 × 10^9^/L
Basophils	0.06	0.09	< 0.1 × 10^9^/L

**Table 2:** The CBC of Case 1 and 2 1 month after antimalarial treatment.

CBC	Case 1	Case 2	Reference range
White blood cells	5.9	5.6	4.0–11.0 × 10^9^/L
Red blood cells	4.8	5.1	4.5–6.5 × 10^12^/L
Hemoglobin concentration	12.8	14.0	12.5–18.0 g/dL
Hematocrit	40.8	44.1	40%–52%
Mean cell volume	85	86.4	78–100 fL
Mean cell hemoglobin	26.6	27.4	27–32 pg
Mean cell hemoglobin concentration	31.3	31.7	31–37 g/dL
Platelets	146	167	150–400 × 10^9^/L
Neutrophils	3.4	3.3	2–7.5 × 10^9^/L
Lymphocytes	2.1	1.8	1–4.5 × 10^9^/L
Monocytes	0.2	0.3	0.2–0.8 × 10^9^/L
Eosinophils	0.15	0.18	0.04–0.40 × 10^9^/L
Basophils	0.05	0.02	< 0.1 × 10^9^/L

## Data Availability

The data supporting this study's findings are available from the corresponding author upon reasonable request.
